# Parental Awareness Towards Antibiotic Use for Upper Respiratory Tract Infections in Children

**DOI:** 10.7759/cureus.79525

**Published:** 2025-02-23

**Authors:** Rima Shah, Siddhartha Dutta, Shubha Singhal, Pinky Meena, Aditya Kadeval

**Affiliations:** 1 Department of Pharmacology, All India Institute of Medical Sciences, Rajkot, IND; 2 Department of Pediatrics, All India Institute of Medical Sciences, Rajkot, IND; 3 Department of Emergency Medicine, Referral Hospital and Community Health Center, Rajkot, IND

**Keywords:** antibiotics, antimicrobial resistance, antimicrobial usage, attitude, knowledge, knowledge- attitude- practices (kap), pediatrics, practices (kap), upper respiratory tract infections (urti)

## Abstract

Background

A common ineffective practice in pediatric care is the prescribing of antibiotics for upper respiratory tract infections (URTIs), which are typically caused by viruses. Behaviors of community members and their knowledge about antibiotic use is one of the major and modifiable factors contributing to antimicrobial resistance (AMR).

Objectives

The aim of this study was to assess the knowledge, attitude, and practices of antibiotic use among parents for URTIs in pediatric patients.

Methodology

This cross-sectional questionnaire-based study included 384 parents of children. Their demographic data and knowledge, attitude, and practices regarding the use of antibiotics in URTI in pediatric patients and self-medication were evaluated using a structured validated 5-point Likert scale-based questionnaire by interviewing them and analyzed using appropriate statistical tests.

Results

Out of 384 participants, 191 (49.74%) were between 30 and 40 years of age, with a male preponderance, 228 (59.38%). Of the participants, 205 (53.39%) did not know whether antibiotics should be given to all children who develop fever, 228 (59.38%) agreed that most URTIs are self-limited, and 222 (57.8%) were unaware of the side effects of antibiotics. Overall, 209 (54.4%) parents would not request an antibiotic prescription from a doctor and 80 (20.9%) parents changed the pediatrician if they prescribed antibiotic at each visit for their child; 337 (87.7%) parents stated that they strictly follow their doctors’ recommendations for the use of antibiotics, 142 (36.97%) parents practice self-medication with antibiotics, the most common source of information was previous prescription, 259 (67.45%), and the most frequent site for procurement was pharmacy store, 275 (71.6%). Major reasons identified for self-medication were perception of URTI as a very simple problem not requiring doctor consultation, 124 (32.4%), followed by unaffordability of costs for a doctor visit and treatment 70 (18.2%). Scores of knowledge about antibiotic use in URTI were significantly associated with parents’ age, education level, and socioeconomic class (p<0.05), while scores of attitude were only significantly associated with socioeconomic class (p<0.5). Practices towards antibiotic use were found to be significantly associated with socioeconomic class and number of children (p<0.05).

Conclusion

Findings of the study show that there is a lack of proper knowledge, attitude, and practices for the use of antimicrobials among parents for URTIs in children. Awareness programs targeting parents for the use of antibiotics and legislative actions for the sale of antibiotics together can help in improving rational use of medicines and contribute to the prevention of AMR.

## Introduction

Upper respiratory infections (URTIs) are common in children. URTI is an acute condition that can vary from a mild, self-limiting illness to a potentially life-threatening disease. It is a leading cause of missed school and unnecessary medical healthcare, which require a great cost burden on both society and healthcare facilities. Majority of the URTI patients are evaluated in outpatient settings [1]. Most cases usually only require reassurance, education, and symptomatic treatment [2,3]. A common ineffective practice in pediatric settings is prescribing antibiotics for URTIs, which are mostly viral in origin [4-6]. However, even certain bacterial infections, such as otitis media and sinusitis, are typically self-limiting, making antibiotic treatment unnecessary. Among many other factors, behaviors of community members and their limited knowledge associated with inappropriate antibiotic use are contributing to antibiotic resistance. Parental beliefs and expectations are important factors in determining whether an antibiotic is prescribed. When parents panic about acute illness, it leads to more frequent pediatric physician visits for URTIs and, subsequently, unnecessary antibiotic use. Parents are not fully aware of the indications and complications of using over-the-counter antibiotics. In the past decade, the rise of antimicrobial resistance (AMR) has become a significant public health concern, as the development of new antibiotics is not advancing quickly enough to keep up with the spread of highly resistant bacteria [7]. In 2019, AMR was directly responsible for an estimated 1.27 million deaths worldwide, surpassing fatalities from HIV/AIDS or malaria, while the development of new antibiotics has drastically slowed, with only 15 new antibiotics approved between 2000 and 2018 compared to 63 between 1980 and 2000 [8].

The American Academy of Pediatrics has provided three fundamental guidelines for the proper use of antibiotics in treating pediatric URTIs: ensuring accurate diagnosis, assessing the risks and benefits, and identifying situations where antibiotics are not appropriate [9]. In line with the WHO's identification of AMR as a critical global issue, India launched its National Action Plan (NAP) on AMR in April 2017 [10,11].

The issue is that the lack of sufficient research and paucity of data not only hampers the estimation of exact rise and extent of AMR in India but also prevents a nationwide comparison. Unnecessary antibiotic prescriptions are a key driver of AMR [12], with both healthcare providers and parents playing a role in this issue [13,14]. While the exact reasons behind the overuse of antibiotics are not fully understood, we developed a knowledge, attitude, and practice questionnaire for parents regarding antibiotic use for URTIs in India. This tool aims to identify the factors contributing to the problem so that targeted educational interventions can be implemented to raise awareness and reduce antibiotic misuse.

Therefore, this study has been planned with the objective of assessing the knowledge, attitude, and practice of antibiotic use among parents for upper respiratory tract infections (URTIs) in pediatric patients.

## Materials and methods

This was a cross-sectional, observational, single-center study conducted in various outpatient departments (OPDs) of a tertiary care teaching hospital in West India to assess the knowledge, attitude, and practices of patients towards antibiotic use in pediatric patients for URTI. The study protocol was presented and approved by an Institutional Ethics Committee (IEC), and participants were explained clearly about the nature and purpose of the study in the language they understood. Written informed consent was obtained before enrolling the participant for the study.

Participant enrollment

Parents of children who visited the pediatric department or any other clinical department at any time after childbirth for preventive care, treatment, or follow-up of URTI and who were able to understand and complete the questionnaire were randomly selected for the study if they had visited any OPD at All India Institute of Medical Sciences, Rajkot (AIIMS), Rajkot, Gujrat, India, during April and May 2023. Parents with severe cognitive impairments, those with known psychiatric conditions, or those unwilling to participate were excluded from the study.

Sample size

The study aimed to assess the prevalence of knowledge about antimicrobial drug use in pediatric URTIs, with approximately 50% of parents expected to have knowledge on the topic. Using a standard deviation (SD) of 9.6, an alpha error of 0.05, a critical difference of 1.90, and a study power of 90%, it was determined that 354 parents would be required for the study. Given that the study was questionnaire-based and factoring in a 10% noncompliance and dropout rate, a total of 384 parents were enrolled.

Study procedure

A total of 384 parents who met the inclusion and exclusion criteria and attended any OPD of AIIMS, Rajkot were enrolled for the study. Parents were approached after they finished a consultation with the doctor in the hospital. All necessary information such as demographic data, history of illness, clinical data, and drug treatment were collected by reviewing the hospital case file and interviewing the patients. All the information gathered was recorded in the structured case record form, and knowledge, attitude, and practices about antibiotic use were evaluated by a questionnaire.

Study tool

A self-structured, validated, and translated questionnaire was developed. To accomplish this goal, literature, standard textbooks, manuscripts, and published papers describing similar research and methodological issues were studied. To assure clarity, accuracy, and consistency of the questions, the questionnaire was pre-tested and evaluated by the scientific team and was also validated by two subject experts. Cronbach’s alpha value for the questionnaire in the study was found to be 0.8.

The knowledge, attitude, and practice questionnaire, aside from gathering demographic information, was organized into three main domains: (1) knowledge, which assessed parents' understanding of issues related to URTIs and antibiotic use, (2) attitude, which explored parents' feelings, beliefs, or perceptions regarding the use of antibiotics for URTIs, (3) practice concerning the ways in which parents demonstrate their knowledge and attitude through their actions. Questions were given options of “strongly agree,” “agree,” “neutral/don’t know,” “disagree,” and “strongly disagree” on a 5-point Likert scale. Each correct response was given 1 point, and for each incorrect response, 1 point was deducted. For “neutral or don’t know” category, zero point was given. Total score of knowledge, attitude, and practice ranged from -9 to +9, -8 to +8, and -6 to +6, respectively.

Statistical analysis

The data collected were entered into an Excel spreadsheet and analyzed using Epi Info software. They were presented as actual frequencies, means, percentages, and SDs, depending on what was appropriate. The chi-square test was applied for qualitative data, while t-tests and one-way ANOVA were used for quantitative data to assess correlation. A p-value of <0.05 was considered statistically significant. Regression analysis was conducted to identify demographic factors associated with knowledge, attitudes, and practices. For the knowledge and attitude questions, a score of "1" was given for correct responses and "0" for incorrect or uncertain answers, with scores summed for each domain. For the practice-related questions, a 5-point Likert scale was used, where responses ranged from "5" (most appropriate) to "1" (least appropriate), and scores were totaled accordingly.

Data management and confidentiality were prioritized: the data were classified and encoded with a unique identification number in the Excel database. It was stored on a password-protected device, accessible only by the principal investigator and the corresponding investigator, and all information was kept confidential. Only the research team had access to the database for analysis purposes.

Ethical considerations

The study protocol was presented to the IEC and permission was obtained before commencement of the study via approval letter no. O.W.No./AIIMS Rajkot/IEC/09/2022 for departmental non-funded project NF/08/2022. This study does not involve any intervention or invasive procedures. Informed consent form was signed by each and every participant if they agree to participate in the study. Assurance of not disclosing their name and details at any stage of study was given.

## Results

Out of the 384 study participants, majority, i.e., 191 (49.74%), were between 30 and 40 years of age, 164 (42.71%) were more than 40 years of age, and only 29 (7.55%) were between 20 and 30 years of age. Table 1 demonstrates the sociodemographic data of the parents. There were 228 (59.38%) male and 156 (40.62%) female respondents in this study group. The difference between age group and sex was found to be statistically significant (p<0.05). Most of them, i.e., 329 (85.68%), had one or two children. Participants’ socioeconomic status was classified into three categories: lower, middle, and upper. Overall, 236 (61.46%) respondents belonged to the middle socioeconomic status, 100 (26.04%) belonged to the upper socioeconomic status, and only 48 (12.5%) belonged to the lower socioeconomic status. This difference among socioeconomic statuses was found to be statistically significant. On analyzing the educational status, it was found that most of the study participants, i.e., 193 (50.26%), had education up to 10th standard, while 113 (29.43%) were graduated, 40 (10.42%) had education above that, and 38 (9.9%) were illiterate. This difference in educational status was found to be statistically significant. As shown in Table 1, only 25 (6.51%) were single parents. The difference between the participant being a single parent or not and gender was found to be statistically insignificant.

**Table 1:** Demographic characteristics of study participants (n=384) *P<0.05 is considered significant

		Male (%)	Female (%)	Total (%)	P-Value
Age (in years)	20 to 30	14 (6.14)	15 (9.62)	29 (7.55)	<0.001*
	30 to 40	86 (37.72)	105 (67.31)	191 (49.74)	
	Above 40	128 (56.14)	36 (23.08)	164 (42.71)	
Socioeconomic status	Lower	30 (13.16)	18 (11.54)	48 (12.50)	0.003*
	Middle	153 (67.11)	83 (53.21)	236 (61.46)	
	Upper	45 (19.74)	55 (35.26)	100 (26.04)	
Education	Illiterate	23 (10.09)	15 (9.62)	38 (9.9)	0.012*
	Upto 10th std	116 (50.88)	77 (49.36)	193 (50.26)	
	Graduate	57 (25)	56(35.9)	113 (29.43)	
	Postgraduate	32 (14.04)	8 (5.13)	40 (10.42)	
Single parent	Yes	17 (7.46)	8 (5.13)	25 (6.51)	0.4
	No	211 (92.54)	148 (94.87)	359 (93.49)	
No. of children	1	74 (32.46)	47 (30.13)	121 (31.51)	0.9
	2	121 (53.07)	87 (55.77)	208 (54.17)	
	3	22 (9.65)	13 (8.33)	35 (9.11)	
	4	11 (4.82)	9 (5.77)	20 (5.21)	

Assessment of knowledge

The majority of parents, i.e., 205 (53.39%), did not know whether antibiotics should be given to children who develop fever or not. A total of 228 (59.38%) of respondents agreed that most URTIs are self-limited without antibiotic treatment. Moreover, 236 (61.5%) did not know that antibiotics would speed the time of recovery from flu symptoms, whereas 95 (24.75%) believed that flu-like symptoms are relieved faster when antibiotics are given. Around 236 (61.5%) parents were able to give the correct answer that "cough is a protective reflex and it should not always be suppressed." However, 222 (57.8%) parents did not know about the side effects of antibiotics, 277 (72.14%) parents did not know that antibiotics can prevent complications from URTI, and 279 (72.66%) parents did not know that inappropriate use of antibiotics can develop resistance to them. The majority of parents, i.e., 255 (66.4%), were aware that antibiotics should not be used without doctors’ advice and 227 (59.1%) parents knew that antibiotics should not be stopped after symptomatic improvement and the whole course should be completed (Table 2).

**Table 2:** Parents' responses (%) to questions related to knowledge with calculated mean score (n=384) The data are represented as N (%) URTI, upper respiratory tract infection

Knowledge		Strongly Agree (%)	Agree (%)	Neutral/don’t know (%)	Disagree (%)	Strongly disagree (%)	Mean score	SD
Q1	Antibiotics should be given to all children who develop fever	14 (3.65)	43 (11.20)	205 (53.39)	106 (27.60)	16 (4.17)	3.17	0.820
Q2	Most URTI are self-limited	22 (5.73)	206 (53.65)	42 (10.94)	100 (26.04)	14 (3.65)	2.68	1.036
Q3	Child with flu-like symptoms get better faster when prescribed antibiotics	13 (3.39)	82 (21.35)	236 (61.46)	45 (11.72)	8 (2.08)	2.88	0.732
Q4	Cough is a protective reflex and it should not be suppressed always	22 (5.73)	214 (55.73)	109 (28.4)	24 (6.25)	15 (3.9)	2.47	0.851
Q5	Antibiotics do not have any side effects	18 (4.7)	65 (16.9)	222 (57.8)	63 (16.4)	16 (4.2)	2.98	0.830
Q6	Antibiotics can prevent complications from URTI	22 (5.7)	57 (14.84)	277 (72.14)	22 (5.7)	6 (1.6)	2.83	0.684
Q7	Inappropriate use of antibiotics reduces their efficacy and causes bacterial resistance	12 (3.13)	73 (19.01)	279 (72.66)	6 (1.56)	14 (3.65)	2.84	0.671
Q8	Antibiotics should not be used without doctors’ advice	61 (15.9)	194 (50.5)	97 (25.3)	22 (5.7)	10 (2.6)	2.29	0.892
Q9	Antibiotics should not be stopped after symptomatic improvement and the whole course should be completed	57 (14.8)	170 (44.3)	93 (24.2)	54 (14.1)	10 (2.6)	2.45	0.992

Assessment of attitude

The majority of parents, i.e., 274 (71.4%), did not know whether antibiotics are used too much and unnecessarily or not. Furthermore, only 80 (20.9%) parents looked for another pediatrician if they were always prescribing antibiotics at each visit, whereas 157 (41%) parents would not change pediatricians even if they were prescribing antibiotics. However, 209 (54.4%) parents would not request an antibiotic prescription if their child suffers from frequent URTI. Around 207 (53.9%) parents were not ready to reuse any leftover antibiotics whenever their child got similar symptoms of a URTI. Around 169 (44%) parents thought that parents and pediatricians should be informed about the judicious use of antibiotics. Table 3 shows that 239 (62.3%) parents agreed to visit the pediatrician even if their child only suffered from a running nose. The majority of parents, 317 (82.6%), would visit a pediatrician to prevent complications of their child’s URTI.

**Table 3:** Parents' responses (%) to questions related to attitude with calculated mean score (n=384) The data are represented as N (%) URTI, upper respiratory tract infection

Attitude		Strongly agree	Agree	Neutral/don’t know	Disagree	Strongly disagree	Mean score	SD
Q1	Do you believe that antibiotics are used too much and unnecessarily?	16 (4.2)	46 (12)	274 (71.35)	36 (9.4)	12 (3.1)	2.95	0.710
Q2	Would you change your pediatrician because whenever you visit him/her, he/she keeps prescribing antibiotics?	18 (4.7)	62 (16.2)	135 (35.2)	157 (40.9)	12 (3.1)	3.22	0.916
Q3	Would you change your pediatrician for not prescribing as many antibiotics as you think he/she should?	4 (1.04)	24 (6.3)	147 (38.3)	187 (48.7)	22 (5.7)	3.52	0.744
Q4	Would you reuse any leftover antibiotics whenever your child gets similar symptoms of a URTI	10 (2.6)	61 (15.9)	106 (27.6)	155 (40.4)	52 (13.5)	3.46	0.998
Q5	Do you think that parents and pediatricians should be informed about the judicious use of antibiotics	43 (11.2)	126 (32.8)	188 (49)	19 (5)	8 (2.1)	2.54	0.836
Q6	Would you request an antibiotic prescription if your child suffers from frequent URTI?	12 (3.1)	18 (4.7)	145 (37.8)	189 (49.2)	20 (5.2)	3.49	0.798
Q7	Do you visit your pediatrician if your child suffers just from running nose?	24 (6.3)	215 (56)	14 (3.7)	121 (31.5)	10 (2.6)	2.68	1.064
Q8	Would you visit a pediatrician in order to prevent any complication of your child’s URTI?	43 (11.2)	274 (71.4)	29 (7.6)	34 (8.9)	4 (1.04)	2.17	0.779

Assessment of practice

Approximately 227 (61.8%) parents often asked their doctors whether or not the prescription of antibiotics was necessary, and more than half of them, 248 (64.7%), mentioned that their doctors never suggested antibiotics over the phone. Moreover, 227 (59.1%) parents sometimes or never asked the doctors to prescribe an antibiotic for their children. In addition, 337 (87.7%) parents stated that they strictly follow their doctor’s instructions and recommendations. The majority of parents, 231 (60.2%), were informed by their doctors about their child’s disease and notified whether it was necessary or not to use antibiotics (Table 4).

**Table 4:** Parents' responses (%) to questions related to practice with calculated mean score (n=384) The data are represented as N (%)

Practice		Always	Most of the times	Often	Sometimes	Never	Mean score	SD
Q1	How often do you ask your pediatrician whether or not the prescription of antibiotics is necessary?	16 (4.2)	61 (15.9)	160 (41.7)	135 (35.2)	12 (3.1)	3.17	0.880
Q2	How often does your pediatrician recommend antibiotics on the phone?	4 (1.04)	25 (6.5)	107 (27.9)	218 (56.8)	30 (7.9)	3.64	0.763
Q3	How often do you ask directly your pediatrician to prescribe antibiotics?	2 (0.5)	28 (7.3)	127 (33.1)	201 (52.3)	26 (6.8)	3.58	0.747
Q4	How often do you completely follow all the pediatrician’s instructions and advice?	108 (28.1)	229 (59.6)	20 (5.2)	19 (4.9)	8 (2.1)	1.93	0.846
Q5	How often do you insist your pediatrician to prescribe antibiotics as a precaution even if the diagnosis is not confirmed?	2 (0.5)	23 (6)	204 (53.1)	117 (30.5)	38 (9.9)	3.43	0.772
Q6	How often does your pediatrician inform you about your child’s disease and notify you whether it is necessary or not to use antibiotics?	28 (7.3)	203 (52.9)	115 (29.9)	24 (6.3)	14 (3.7)	2.46	0.860

Association of score of knowledge, attitude, and practices with various demographic parameters

Table 5 shows the association of mean scores of the knowledge, attitude, and practices with different demographic parameters. Knowledge about antibiotic use in URTI was significantly associated with parents’ age, education level, and socioeconomic class (p<0.05), while score of attitude was only significantly associated with socioeconomic class (p<0.5). Practices towards antibiotic use were found to be significantly associated with socioeconomic class and number of children (p<0.05).

**Table 5:** Correlation between sociodemographic characteristics and score of knowledge, attitude, and practices toward antibiotic use in URTI (n=384) Results are expressed as the mean ± standard deviation The p-values have been calculated using independent‑test and one‑way ANOVA. **Significant at p≤0.05 URTI, upper respiratory tract infection

Factor	Average knowledge score (out of 9)	Average attitude score (out of 8)	Average practice score (out of 6)
Gender			
Male	2.1±2.4	1.95±1.98	2.61±1.6
Female	2.24±2.36	1.85±2.0	2.45±1.68
P-value	0.603	0.633	0.358
Age group in years			
20-30 years	2.71±0.76	1.81±0.53	2.8±0.6
30-40 years	1.78±0.34	1.81±0.28	2.6±0.23
>40 years	2.5±0.37	2.04±0.33	2.43±0.26
P-value	0.008	0.539	0.431
Educational level			
Illiterate	0.85±1.4	1.26±1.7	2.26±1.5
Upto 10th Std.	2.13±2.3	2.1±1.9	2.42±1.6
Graduate	2.74±2.2	1.8±1.9	2.81±1.75
Postgraduate	2.26±1.95	2.68±2.05	2.67±1.6
P-value	0.000**	0.094	0.145
Income level			
Low	1.73±1.77	2.1±1.8	2.4±1.6
Middle	2.5±2.2	2.3±1.9	2.75±1.5
Upper	2.9±1.7	1.8±0.95	2.8±1.85
P-value	0.015	0.000**	0.027
Number of children			
1 child	2.34±2.3	2.0±1.9	2.63±1.7
2 children	2.1±2.4	1.95±2.08	2.61±1.54
3 children	2.6±2.1	1.9±1.82	1.6±1.7
4 children	2.51±1	1.95±1.4	3.1±1.41
P-value	0.082	0.699	0.001

**Figure 1:**
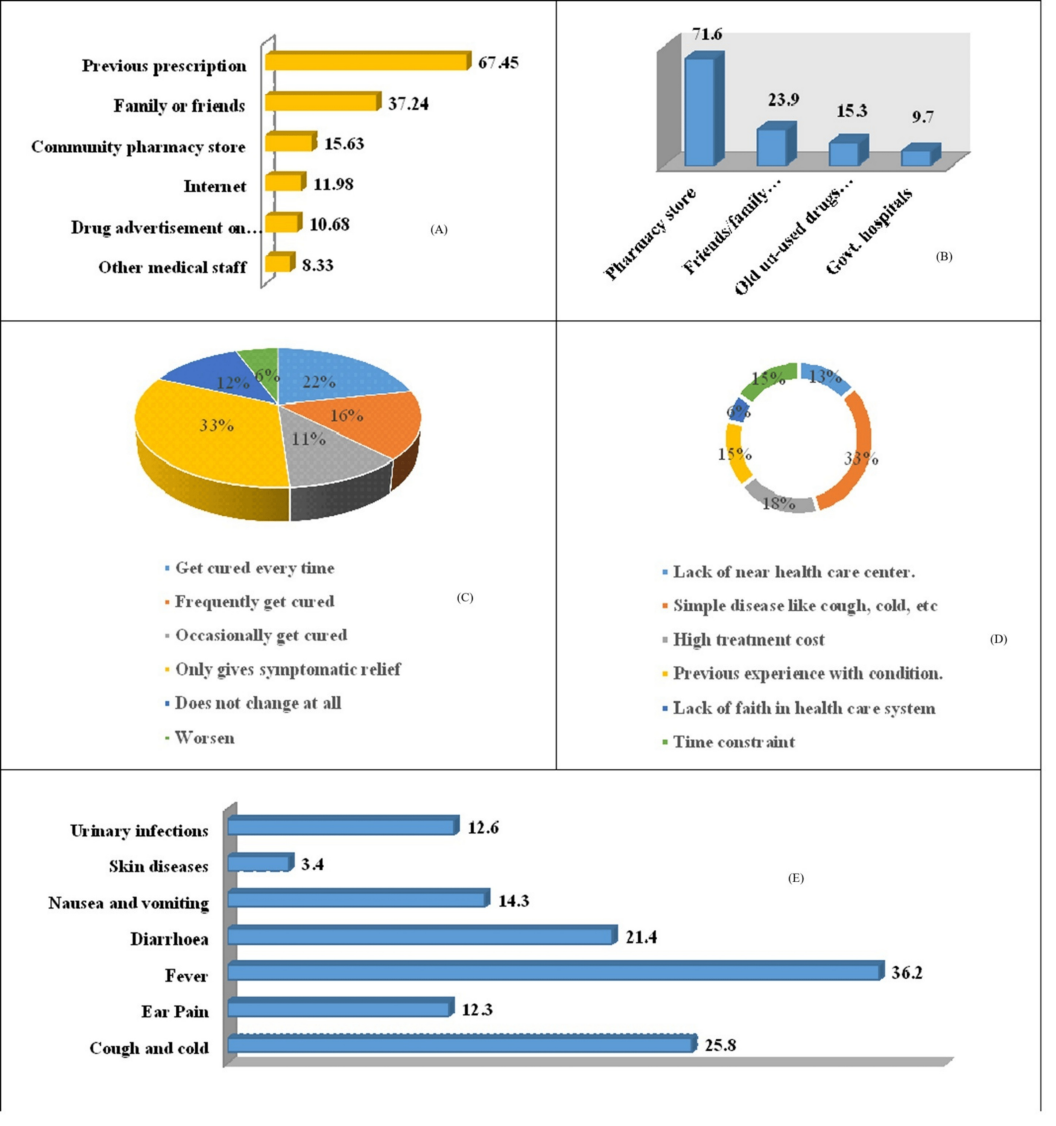
Assessment of self-medication practices for antimicrobial drugs among parents (A) Different sources of information for self-medication of antimicrobial agents. (B) Analysis of place of procurement of antibiotic for self-medication. (C) Analysis of outcome of disease/symptoms after antibiotic self-medication. (D) Reasons for self-medication of antibiotics. (E) various diseases/symptoms for which self-medication was taken.

Assessment of self-medication of antimicrobial drugs by parents is shown in Figure 1. The previous prescription by doctors was chosen as the major source of information, i.e., 259 (67.45%) for self-medicating antibiotics, followed by family or friends, 143 (37.24%), pharmacist, 60 (15.63%), internet, 46 (11.98%), both mass media and TV, 41 (10.68 %), , and other medical staff, 32 (8.3%). Figure 1a displays the sources of information which parents turned to. Figure 1b depicts that the majority of parents, i.e., 275 (71.6%), purchase antibiotics from a pharmacy store, followed by 92 (23.9%) from friends and family, whereas only 59 (15.3%) used old medicines at home. The majority of parents 126 (32.9%) experienced only symptomatic relief from self-medicating their child with antibiotics, whereas in 84 (21.7%) of cases, it was cured every time, and in 60 (15.8%) of cases, the disease outcome was frequently cured. In only 22 (5.7%) of cases, the situation worsened (Figure 1c). Figure 1d shows that the main reason for self-medication by parents for their child, 124 (32.4%), was a simple disease such as cough or cold; however, 70 (18.2%) parents do it because of the high treatment cost, followed by 58 (15%) due to time constraints and previous experience with similar conditions. Only 23 (6.1%) parents lack faith in the healthcare system. Most commonly, antibiotics are used for fever, 139 (36.2%), followed by cough and cold, 99 (25.8%), diarrhea, 82 (21.4%), nausea and vomiting, 55 (14.3%), urinary infection, 48 (12.6%), ear pain, 47 (12.3%), and skin disease, 13 (3.4%) (Figure 1e).

## Discussion

In the current era of antibiotic resistance and irrational use of antibiotics, especially in the pediatric population during URTIs, several factors contribute to this issue, including limited knowledge among parents regarding the indications and complications of over-the-counter antibiotics and the common ineffective practice of prescribing antibiotics for URTIs, which are mostly viral in origin. There has been a growing concern surrounding antibiotic resistance as it is a significant global health threat, leading to increased morbidity, mortality, and healthcare costs [14]. Inappropriate antibiotic use, such as prescribing antibiotics for viral URTIs, contributes to this problem. Parents play a crucial role in determining whether their child receives antibiotics or not for early recovery, and their false beliefs, expectations, and behavior greatly influence the decision to prescribe antibiotics for URTIs in pediatric patients [15].

In the present study, data from 384 participants were examined for various sociodemographic factors, including age group, sex, socioeconomic status, educational status, and single parenthood. The findings of this study revealed that the majority of participants were between 30 and 40 years of age, accounting for 49.74% of the sample. These differences between age groups were found to be statistically significant, suggesting that certain demographic factors may influence knowledge, attitude, and practice regarding antibiotic use for URTIs. This age distribution suggests that parents in the older age groups are more prevalent in the study population. This finding is consistent with previous studies that have reported a higher likelihood of seeking medical care and receiving antibiotics for their children among older parents or caregivers [16]. Regarding gender distribution, the study observed a significantly higher number of male participants (59.38%) compared to females (40.62%). Males usually visit hospitals more as they are handling most outdoor work of any home as per Indian culture, which may contribute to more males in this study. Findings are similar to the previous studies [15,16]. On evaluating the socioeconomic status, it was observed that majority of respondents belonged to the middle socioeconomic status (61.46%), followed by the upper socioeconomic status (26.04%), and a smaller proportion belonged to the lower socioeconomic status (12.5%). This difference in socioeconomic status was statistically significant. These findings suggest that socioeconomic factors may play a role in parents' knowledge, attitude, and practice of antibiotic use. On analyzing the educational status of the participants, majority had education up to the 10th standard (50.26%), followed by graduates (29.43%), those with education above graduation (10.42%), and illiterate individuals (9.9%). This difference in educational status was found to be statistically significant. The findings suggest that higher educational attainment may be associated with a better understanding of appropriate antibiotic use. Similar findings have been reported in previous studies, emphasizing the importance of educational interventions to improve parents' knowledge and promote responsible antibiotic use [17,18].

The study revealed significant knowledge gaps and misconceptions among parents regarding the use of antibiotics for URTIs in pediatric patients. A considerable number of parents were unsure about when to give antibiotics, believed that antibiotics could speed up recovery from viral infections, and were unaware of the side effects and the risk of antibiotic resistance. These findings align with previous studies conducted globally, highlighting the need for targeted educational interventions to enhance parental understanding of appropriate antibiotic use, promote the limited role of antibiotics in viral infections, and emphasize the importance of preserving the effectiveness of antibiotics [19,20]. Addressing these knowledge gaps is crucial in reducing inappropriate antibiotic use and combating the growing threat of antibiotic resistance.

On analyzing the attitudes among parents regarding antibiotic use for pediatric URTIs, a significant proportion of parents lacked awareness about excessive and unnecessary antibiotic use, and many would not consider changing pediatricians even if antibiotics were consistently prescribed. However, some parents recognized the need to avoid requesting antibiotics for frequent URTIs. Furthermore, a substantial number of parents were unwilling to reuse leftover antibiotics, indicating a lack of understanding about appropriate antibiotic use. It is evident that parental attitudes towards antibiotic use in pediatric URTIs vary, and the overall prevalence of inadequate knowledge and misconceptions surrounding antibiotics is consistent with findings of other studies [21].

The study findings also highlight a majority of parents frequently inquire about the necessity of antibiotic prescriptions for pediatric URTIs, demonstrating an active involvement in decision-making. It was positive to note that a significant number of parents did not ask for antibiotics, suggesting a cautious approach towards antibiotic use. Additionally, parents reported high levels of adherence to pediatricians' instructions and recommendations. These findings align with the importance of effective communication, shared decision-making, and trust between parents and healthcare providers [22]. Comparisons with previous studies highlight similar results of active involvement, cautiousness, and trust [22].

The knowledge, attitude, and practice analysis across demographic parameters reveals significant associations, particularly with knowledge and practice domains. Knowledge scores exhibited a statistically significant difference among age groups (p=0.008), with younger participants (20-30 years) demonstrating higher scores (2.71±0.76) compared to older cohorts, suggesting greater awareness and information retention in younger individuals. Educational level was a key determinant of knowledge, with graduates exhibiting the highest scores (2.74±2.2, p=0.000), reinforcing the role of formal education in enhancing cognitive understanding. Similarly, income level significantly influenced knowledge scores (p=0.015), with upper-income individuals displaying superior knowledge, likely due to better access to educational resources and healthcare information. Attitude scores did not significantly differ by gender (p=0.633) or age group (p=0.539), but income levels had a significant impact, indicating that socioeconomic factors may shape perceptions and receptivity toward health-related practices. The practice domain was significantly influenced by income (p=0.027) and the number of children (p=0.001), with individuals from higher-income groups and those with four children exhibiting better adherence to recommended practices, possibly due to greater experience and resource availability. These findings underscore the critical role of socioeconomic and educational factors in shaping health-related knowledge and behaviors, highlighting the necessity for targeted interventions to improve awareness and practice among lower-income, less-educated, and older populations.

The findings regarding the distribution of sources of information and practices related to self-medication with antibiotics among parents are concerning. The study reveals that the previous prescription by doctors is the major source of information for self-medicating antibiotics, indicating a potential misuse of medication without proper evaluation. Additionally, the reliance on family or friends, pharmacists, and the internet as sources of information highlights the influence of informal channels in antibiotic decision-making. Furthermore, the high percentage of parents purchasing antibiotics from pharmacy stores and using old medicines at home raises concerns about the quality and appropriateness of the medication being used. The outcomes of self-medication varied, with a majority of parents experiencing only symptomatic relief, while a significant number reported successful outcomes. However, a notable percentage of cases resulted in a worsening of the situation increasing the risk associated with self-medication [23,24].

This study provides a comprehensive assessment of knowledge, attitudes, and practices regarding antibiotic use among a diverse sample of parents, which is a key strength. The use of validated questionnaires ensures reliable data, and rigorous statistical analysis supports the robustness of the findings. However, there are several limitations, including the single-center design, which limits the generalizability of the results, and the lack of qualitative analysis, which could have provided deeper insights into the beliefs and behaviors influencing antibiotic use. Additionally, the potential for bias in self-reported data exists, as participants may have over- or under-reported their antibiotic use, which could affect accuracy. Despite these limitations, the study offers valuable insights into parental antibiotic use practices, and future research should address these limitations through multi-center designs, qualitative approaches, and more objective measures to enhance the validity and generalizability of findings.

## Conclusions

Findings of the study show that there is a lack of proper knowledge, attitude, and practices for the use of antimicrobials among parents for URTI in children. Also, there is high prevalence of self-medication of antibiotics, which can be harmful for patients and contribute to AMR. Awareness programs targeting the parents for the use of antibiotics and legislative actions for sell of antibiotics together can help in improving the rational use of medicines and contribute to the prevention of AMR.
